# Health Risk Assessment of Heavy Metal(loid)s in the Overlying Water of Small Wetlands Based on Monte Carlo Simulation

**DOI:** 10.3390/toxics12070488

**Published:** 2024-07-03

**Authors:** Liling Wang, Mamattursun Eziz, Yonglong Hu, Xayida Subi

**Affiliations:** 1 College of Geographical Science and Tourism, Xinjiang Normal University, Urumqi 830054, China; wll18152166428@163.com (L.W.); a18048617279@163.com (Y.H.); xayida104@126.com (X.S.); 2 Laboratory of Arid Zone Lake Environment and Resources, Xinjiang Normal University, Urumqi 830054, China

**Keywords:** small wetlands, HMs, contamination, health risk, Monte Carlo simulation

## Abstract

Heavy metal(loid) (HM) contamination is a significant threat to wetland ecosystem. However, contamination risks of HMs in overlying water of small wetlands, which are easily ignored because of their minor occupancy in an overall area, are nearly unknown. A total of 36 water samples containing six HMs were collected from the urban and rural small wetlands of Urumqi in China, and the contamination levels and probabilistic health risks caused by HMs were assessed using the Nemerow pollution index (NPI) and the health risk assessment model introduced by the US EPA. The results revealed that the average concentration of Hg in the urban and rural small wetlands surpassed the Class II thresholds of the Environmental Quality Standards for Surface Water (GB 3838-2002) by factors of 3.2 and 5.0 times, respectively. The overall contamination levels of HMs in the small wetlands fall into the high contamination level. Results of a health risk assessment indicated that non-carcinogenic health risk of the investigated HMs are found to be lower than the acceptable range for adults, but higher than the acceptable range for children. Meanwhile, As falls into the low carcinogenic risk level, whereas Cd falls into the very low carcinogenic risk level. Overall, HMs in rural small wetlands showed relatively higher contamination levels and probabilistic health risks than that of urban small wetlands. In addition, As was identified as the dominant health risk factor in the overlying water of small wetlands in the study area. Findings of this study provide scientific support needed for the prevention of HM contamination of small wetlands in arid zones.

## 1. Introduction

Wetlands are an important and unique component of global ecosystems, known as the “Kidney of the Earth”. They, along with forest ecosystem and ocean ecosystem, are listed as the three main ecosystems on Earth [1]. Wetlands play a crucial role in the environmental cycle and provide many services to humanity [2]. They have multiple important eco-environmental functions such as water supply [3], water purification [4], flood regulation [5], erosion control [6], carbon storage [7], climate regulation [8], and biodiversity protection [9]. Wetlands are also a key component of regional ecological safety. In recent decades, due to extensive anthropogenic activities, wetlands face a severe problem of non-point source pollution. Environmental contaminants, including HMs [10], COD [6], crude oil [11], nitrogen pollutants [12], phthalate esters [13], and microplastics [14] in wetland ecosystems are increasing environmental concern due to their probabilistic health risks.

Small wetlands refer to the natural or manmade wetland ecosystems covering an area smaller than 8 ha, including small lakes, ponds, seasonal ponds, wet depressions, streams, springs, small rivers, and channels [15,16]. In general, small wetlands are easily ignored in assessing landscape function because of their minor occupancy in an overall area. Despite their relatively smaller size in comparison to larger water bodies, small wetlands exhibit greater abundance and richness [17]. Like bigger water bodies, small wetlands also play a significant role in maintaining regional ecological balance, safeguarding fresh water resources [18], mitigating the impacts of climate change and biodiversity [19], regulating biogeochemical cycles [20], and providing essential ecosystem services [21]. Small wetlands are very sensitive to regional hydrological conditions and can obviously effect the structures and functions of ecological networks [15]. Furthermore, small wetlands contribute notably to global CO_2_ and CH_4_ diffusion emissions [22]. Moreover, elevated organic carbon burial rates are observed in small lakes and reservoirs compared to larger ones, with small reservoirs responsible for a significant portion of nitrogen removal [23]. However, while larger wetlands such as lakes, reservoirs, and rivers have attracted much attention, small wetlands have received comparatively less attention [24]. During the COP13 (The 13th Meeting of the Conference of the Contracting Parties to the Ramsar Convention on Wetlands) in 2018, the “Resolution on Conservation and Management of Small Wetlands”, which was drafted by China, was successfully passed, and the ecosystem functions of small wetlands have received more attention from the international community since. In March 2023, the Chinese National Standard for “Specification on the conservation and management of small wetlands, (GB/T 42481-2023) [25]” was officially released, which standardized the investigation, registration, and restoration of small wetlands.

Arid zone small wetlands play a pivotal role in preserving the ecosystem health and stability of arid regions and are crucial for human survival and development. In arid zones, small wetlands are important water bodies in storing and regulating water resources through distinct hydrological processes. Serving as reservoirs during dry seasons, they mitigate water scarcity and provide essential irrigation water for surrounding ecosystems and agriculture [26]. Arid zone small wetlands can offer a consistent and stable water supply for plants and animals and foster biodiversity preservation [27]. Additionally, they also play a pivotal role in carbon storage within arid ecosystems [7]. Through vegetation growth and organic matter deposition, they also contribute to climate change mitigation [28]. Furthermore, arid zone small wetlands have the capacity to prevent and alleviate drought-related disasters in arid areas. They can improve soil moisture, and protect local agriculture and natural ecosystems from adverse effects of drought climate [29]. Furthermore, arid zone small wetlands also can support industries, thereby providing essential livelihood opportunities for local residents. Therefore, the socioeconomic roles of arid zone small wetlands cannot be overlooked.

In recent years, the ecological safety of small wetlands has been degraded due to intensive anthropogenic activities and climate change [15,30]. Contamination of small wetlands has always posed a serious challenge to water quality management. Among the various contaminants in water environments, HMs are a crucial indicator of wetland contamination. HMs are harmful environmental contaminants due to their high toxicity, non-degradability, and bioaccumulation [31,32]. Therefore, water contamination by HMs is a global concern due to its probabilistic risks to human beings, animals, and plants [33,34]. Currently, major global water systems face significant challenges due to HM contamination, which make it one of the most urgent environmental issues worldwide, particularly concerning surface water contamination [35]. Therefore, numerous researchers have focused on the contamination risks of HMs in larger water bodies, including lakes [36], reservoirs [37], rivers [38], wetlands [10], and groundwater [32]. However, the contamination risks of HMs in small wetlands are nearly unknown. HMs in small wetlands not only affect the function of the wetland ecosystem, but also seriously threaten human health through the water cycle. Therefore, investigation of HMs in the overlying water of small wetlands, especially in arid zones, is needed to identify their potential risks.

The objectives of this work are to (1) identify the concentrations of six HMs, including Hg, As, Cu, Zn, Pb, and Cd, in the overlying water of small wetlands in the Urumqi, northwest arid zones of China; (2) assess the water quality based on the Nemerow pollution index; (3) quantify potential human health risks of HMs using the US EPA health risk assessment model; and (4) compare contamination risks of HMs between urban and rural small wetlands. The obtained results can provide basic data for understanding contamination risk of small wetlands, and prove helpful for ensuring ecosystem security of small wetlands in arid zones.

## 2. Materials and Methods

### 2.1. Study Area and Water Sampling

**Figure 1:**
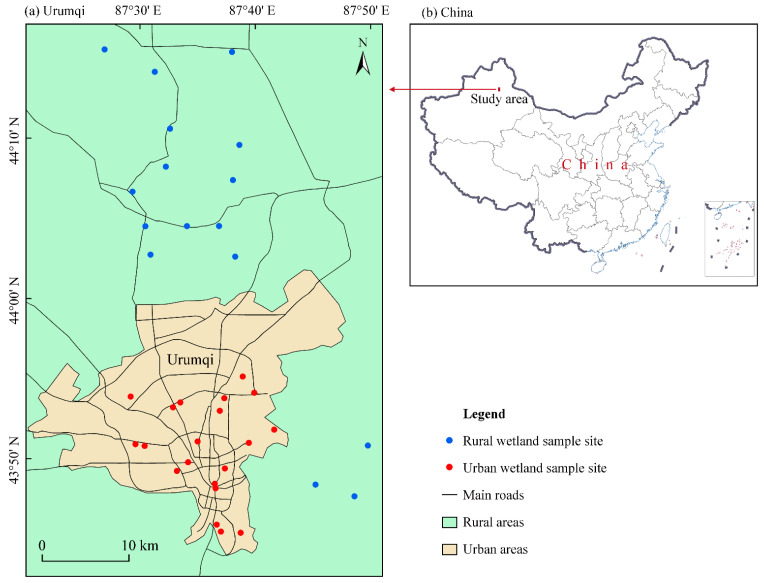
Map of the location of the study area and sample sites.

Field research was conducted in the Urumqi city in the northwest arid zones of China. The Urumqi is the second largest city in NW China and has emerged as an area of increasing economic importance in the Silk Road Economic Belt. The geographic coordinates of the study area are 87°19′–87°54′ E and 43°35′–44°17′ N. It located in the northern piedmont plains of the Tianshan Mountains, and at the southern edge of the Junghar Basin (Figure 1), at altitudes ranging between 680 m and 950 m. The study area has a typical continental desert climate, with an average annual temperature, precipitation, and evaporation of 6.7 °C, 280 mm, and 2730 mm, respectively [39].

A total of 36 samples from the overlying water of small wetlands were collected in October 2023, as illustrated on the map in Figure 1. All samples were collected from small lakes or ponds in the study area. One sample was collected from each small wetland. Among them, 20 samples were collected from urban areas, while 16 samples were collected from rural areas. The collected samples were stored in 500 mL polyethylene bottles. Before sampling, each polyethylene bottle was rinsed three times with the sampling water before being finally filled. Then, 65% HNO_3_ (Anyan, China) was added to each water sample until reached a pH < 2, followed by immediate transportation to laboratory, where they were sealed and stored at 4 °C. In the laboratory, the collected water samples from the small wetlands were all syringe-filtered using a 10 mL disposable syringe (0.45 µm filter pore size), then stored in 50 mL polypropylene bottles.

### 2.2. Sample Analysis

The concentrations of Hg, As, Cu, Zn, Pb, and Cd were determined based on the GB/T 5750.6-2006 [40]. The atomic fluorescence spectrophotometer (BAF–4000, Baode, Beijing, China) was used for measuring concentrations of As and Hg, while inductively coupled plasma–mass spectrometer (ICP–MS, Perkin Elmer, Waltham, MA, USA) was used for measuring concentrations of Cu, Zn, Pb, and Cd. The detection limits for Hg, As, Cu, Zn, Pb, and Cd are 0.04, 0.30, 0.08, 0.67, 0.09, and 0.05 μg/L, respectively. Calibration curves were produced, using quality control standards, for assessing data from each set of water samples. Concentrations of HMs in water samples were tested three times, and the average value was taken as the final concentration. The relative standard deviation (RSD) for the test substances was maintained below 15%. The recovery rates for all targeted HMs ranged from 94.3% to 106.8%.

### 2.3. Contamination Assessment

The Nemerow pollution index (NPI) [41] is used to comprehensively characterize the individual and overall contamination levels of HMs in the overlying water of small wetlands. It calculated as follows:

(1) *P_i_* = *C_i_*/*S_i_*

(2) $$\mathrm{NPI}=\sqrt{\left({P_{max}}^{2}+{P_{ave}}^{2}\right)\;/\;2}$$

where *P_i_* is the contamination index for a single metal *i*, *C_i_* represents the concentration of metal *i* (μg/L), and *S_i_* represents the evaluation standard of metal *i* (μg/L). The Environmental Quality Standards for Surface Water (GB 3838-2002) introduced by the MEEPRC (Ministry of Ecology and Environment of the People’s Republic of China) is selected as the evaluation standard [42]. NPI represents the contamination index caused by the overall contamination by all investigated HMs, and *P_max_* and *P_ave_* are the maximum and average value of *P_i_*, respectively. The classification standard for the contamination degree of *P_i_* and NPI are shown in Table 1 [32,41].

### 2.4. Health Risk Assessment

Oral ingestion and dermal absorption of water are two main routes of exposure of HMs in small wetlands. The exposure of HMs in the small wetlands was characterized by the chronic daily intake (CDI, mg/kg/d) suggested by the US EPA [43]. The populations were divided adults and children. The CDI values for the two exposure routes are computed using the following equations:

(3) $${\mathrm{CDI}}_{i,j,ing}=(C_{i,j}\;\times \;IR\;\times \;EF\;\times \;ED)\;/\;(BW\;\times \;AT)$$

(4) $${\mathrm{CDI}}_{i,j,derm}=(C_{i,j}\;\times \;SA\;\times \;K_{p}\;\times {\;T}_{\mathrm{event}}\;\times \;EV\;\times \;EF\;\times \;ED\;\times {\;10}^{-3})\;/\;(BW\;\times \;AT)\;$$

where *C_i,j_* is the concentration of metal *j* in water sample *i* (μg/L), CDI*_i,j,ing_* represents the chronic daily intake from ingestion route of metal *j* in water sample *i*, and CDI*_i,j,derm_* represents the chronic daily intake from dermal contact route of metal *j* in water sample *i*.

Considering the total non-carcinogenic health risks posed by investigated HMs, the hazard quotient (HQ) and the hazard index (HI) were used.

(5) $$\mathrm{HQ}i,j={(\mathrm{CDI}}_{i,j,\mathrm{ing}}/{\mathrm{RfD}}_{\mathrm{ing}})\;+{(\mathrm{CDI}}_{i,j,\mathrm{derm}}/{\mathrm{RfD}}_{\mathrm{derm}})$$

(6) $$\mathrm{THI}=\Sigma {\mathrm{HQ}}_{i}$$

where HQ*_i,j_* is the hazard quotient for the metal *j* in water sample *i*. *RfD_ing_* and *RfD_derm_* are the reference dose for the ingestion and dermal contact routes, respectively (µg/kg/d). The parameters in CDI estimation are detailed in Table 2. The *RfD* and *SF* values are given in Table 3 [33].

When HQ or HI < 1, HMs in the small wetlands will not have non-carcinogenic risks. HQ or HI > 1 indicates a potential non-carcinogenic health risks [47,48].

The potential carcinogenic health risk of a single metal in the small wetlands is calculated as the cancer risk (CR) as in Equation (7):

(7) $${\mathrm{CR}}_{i,j}={\;\mathrm{CDI}}_{i,j,\mathrm{ing}}\;\times \;SF_{\mathrm{ing}}+{\mathrm{CDI}}_{i,j,\mathrm{derm}}\;\times \;SF_{\mathrm{derm}\;}$$

where CR*^k^_i,j,r_* represents the cancer risk of the metal *j* in the water sample *i*. *SF_ing_* and *SF_dem_* indicate the slope factor for ingestion and dermal contact routes, respectively ((µg/kg/d)^−1^). The *SF* values of HMs are also detailed in Table 3 [33]. The total cancer risk (TCR) posed by all investigated HMs can be determined by summing the estimated cancer risk of each HM and is calculated as follows [49]:

(8) $$\mathrm{TCR}=\Sigma {\mathrm{CR}}_{i}$$

The risk degree of CR or TCR are classified as very low risk (CR or TCR < 1 × 10^−6^), low risk (1 × 10^−6^ ≤ CR or TCR < 1 × 10^−4^), moderate risk (1 × 10^−4^ ≤ CR or TCR < 1 × 10^−3^), high risk (1 × 10^−3^ ≤ CR or TCR < 0.1), and extremely high risk (CR or TCR > 0.1) [46,50].

### 2.5. Monte Carlo Simulation

Health risk assessment involves characteristics such as multiple variables, randomness, and temporal variation, which directly or indirectly contribute to the ambiguity and uncertainty of predictive models [31]. However, the Monte Carlo simulation (MCS) offers more dependable risk assessments by quantifying the probability of surpassing or not surpassing risk thresholds [51]. The MCS is a mathematical theory used for uncertainty analyses in probabilistic health risk assessment. To minimize uncertainty and provide a more robust health risk assessment results, the MCS approach was used to simulate the uncertainty of exposure parameters for health risk assessment. Integrating the US EPA health risk assessment method with Monte Carlo analysis yields probability distributions for HI and TCR values; thus, assessing the uncertainty of the NPI in terms of human health risk exposure [52]. Moreover, sensitivity analysis aids in identifying the most significant health risk factors. Sensitivity is contingent on the correlation coefficient between each CDI estimation parameter and the risk value. A higher correlation coefficient indicates a greater contribution to the final health risk. The Monte Carlo simulation in this study was used to estimate the influences of metal concentrations, *IR*, *ED*, *BW*, and *SA* to identify the sensitivity of the assessment indicators. Related parameters for the health risk assessment model and Monte Carlo simulation are also given in Table 2 and Table 3. Monte Carlo analysis and sensitivity analysis were conducted using Crystal Ball 11.1 (Oracle Inc., Austin, TX, USA).

## 3. Results

### 3.1. The Concentrations of HMs in the Overlying Water of Small Wetlands

The basic statistical summary of concentrations of the investigated HMs in the overlying water of small wetlands and the Class II thresholds of the Environmental Quality Standards for Surface Water (GB 3838-2002) are detailed in Table 4.

As shown there, the average concentrations of Hg, As, Cu, Zn, Pb, and Cd in the urban small wetlands were 0.16 µg/L, 2.70 µg/L, 37.76 µg/L, 93.18 µg/L, 1.20 µg/L, and 0.09 µg/L, respectively. Meanwhile, the average concentrations of these six HMs in the rural small wetlands were 0.25 µg/L, 4.15 µg/L, 63.29 µg/L, 175.65 µg/L, 2.18 µg/L, and 0.14 µg/L, respectively. The average concentration of Hg in the urban and rural small wetlands surpassed the Class II thresholds of the Environmental Quality Standards for Surface Water (GB 3838-2002) by factors of 3.2 and 5.0 times, respectively. The average concentrations of other five HMs were less than the corresponding threshold values. The standard-exceeding ratios (the proportion of samples with concentrations exceeding the thresholds of National Standard to the total number of samples) for Hg in the urban and rural small wetlands were 80.0%, and 81.25%, respectively. Depending on the above analysis, it appears that Hg is particularly more abundant in the overlying water of small wetlands in the study area. It should be noted that the average concentrations of all HMs in the rural small wetlands are higher than those of the urban small wetlands.

The calculated CV (coefficient of variation) values of all HMs in the urban small wetlands and CVs of Hg and Cu in the rural wetlands showed a strong variability (CV > 0.50), indicating high variability for these HMs [31]. Among them, Hg demonstrated the highest variability, reaching 1.29 and 1.25 for the urban and rural small wetlands, respectively. The CVs of As, Zn, Pb, and Cd in the rural small wetlands showed a moderate spatial variability. The pH value of the collected water samples ranged from 6.84 to 8.36, with an average value of 7.43. The electrical conductivity (EC) of the collected water samples ranged from 423.0 μS/cm to 5820.0 μS/cm, with an average value of 1953.94 μS/cm.

### 3.2. Contamination Levels of HMs in the Overlying Water of Small Wetlands

The contamination of HMs in the overlying water of small wetlands was categorized based on the *P_i_* and NPI (Table 5). Depending on the average value of *P_i_*, the contamination degree of HMs in the urban small wetlands decreased in the following order, *P*_Hg_ (3.21) > *P*_Pb_ (0.12) > *P*_Zn_ (0.09) > *P*_As_ (0.05) > *P*_Cu_ (0.04) > *P*_Cd_ (0.02), with greater *P_i_* values showing higher contamination degrees. Meanwhile, the contamination degree of HMs in the rural small wetlands decreased in the following order: *P*_Hg_ (4.98) > *P*_Pb_ (0.22) > *P*_Zn_ (0.18) > *P*_As_ (0.08) > *P*_Cu_ (0.06) > *P*_Cd_ (0.03). Based on the classification standard of *P_i_* and the calculated values of *P_i_* of HMs in the small wetlands, both the urban and rural small wetlands were high contaminated by Hg, whereas other five HMs showed the no contamination level.

The NPI values of HMs in the urban small wetlands ranged from 0.58 to 12.62 with an average value of 2.31, at a moderate contamination level compared to the classification standard of NPI. The NPI values of HMs in the rural small wetlands ranged from 0.58 to 16.22 with an average value of 3.58, at a high contamination level. Overall, the contamination level of HMs in the overlying water of the rural small wetlands was higher than that of the urban small wetlands.

### 3.3. Non-Carcinogenic Health Risk of HMs in the Overlying Water of Small Wetlands

**Figure 2:**
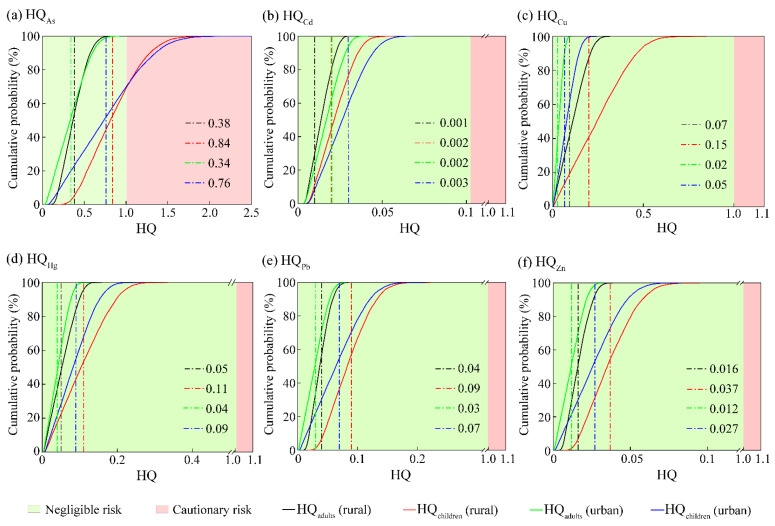
Probability distribution for HQ of HMs in the small wetlands (the black, red, green, and blue vertical dashed lines represented the average HQ values for adult (rural), children (rural), adult (urban), and children (rural), respectively).

Based on Monte Carlo simulation, the HQ of children and adults exposed to HMs in the overlying water of small wetlands through two different exposure routes were assessed, and the probability distribution of HQ is presented in Figure 2. The average HQ values, both children and adults, of HMs in the urban small wetlands decreased in the following order, HQ_As_ > HQ_Hg_ > HQ_Pb_ > HQ_Cu_ > HQ_Zn_ > HQ_Cd_, with the HQ values of all HMs were lower than 1 for adults. But, for children, the maximum HQ of As reached 2.38, and reached 1 when the cumulative frequency reached 70.16%. Conversely, the maximum HQ values for the other five elements consistently remain below 1. Based on the classification standard of HQ, As in the urban small wetlands may pose a potential non-carcinogenic health risk to children, while the non-carcinogenic health risk other five HMs can be ignored. The average HQ values, both children and adults, of HMs in the rural small wetlands decreased in the following order, HQ_As_ > HQ_Cu_ > HQ_Hg_ > HQ_Pb_ > HQ_Zn_ > HQ_Cd_, with the HQ values of all HMs were lower than 1 for adults. But, for children, the maximum HQ of As reached 2.2, and reached 1 when the cumulative frequency reached 69.43%. It indicates that As contributes the most to the potential non-carcinogenic health risks of HMs. The above analysis proves that the HQ of HMs in the small wetlands in the study area was negligible for adults. But As in the small wetlands may pose a potential non-carcinogenic health risk to children. It indicates that children suffered from a higher accumulative non-carcinogenic health risk compared with adults.

**Figure 3:**
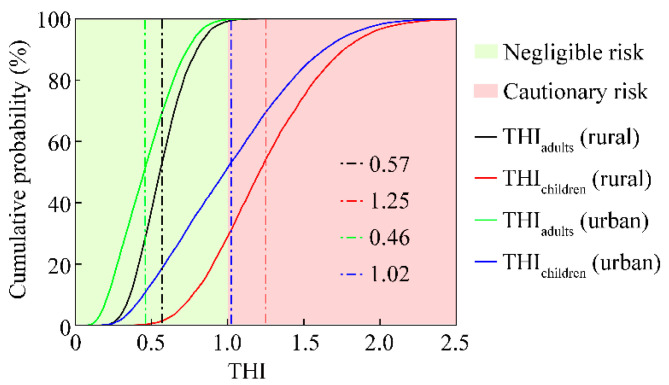
Probability distribution for THI of HMs in the small wetlands (the black, red, green, and blue vertical dashed lines represented the average THI values for adult (rural), children (rural), adult (urban), and children (rural), respectively).

As shown in Figure 3, for adults, the average THI values for the urban and rural small wetlands were 0.46 and 0.57, respectively. It indicates that the total non-carcinogenic risk of HMs in the small wetlands was negligible for adults. For children, the average THI values of HMs in the urban and rural small wetlands were 1.02 and 1.25, respectively, while those for adults were consistently below 1. These findings suggest that HMs in the small wetlands may pose potential non-carcinogenic risk to children, whereas adults generally maintain a safe profile.

Moreover, HMs in the rural small wetlands have relatively higher non-carcinogenic health risk than that of the urban small wetlands. Overall, As was identified the main non-carcinogenic factor in the overlying water of small wetlands in the study area.

### 3.4. Carcinogenic Health Risk of HMs in the Overlying Water of Small Wetlands

**Figure 4:**
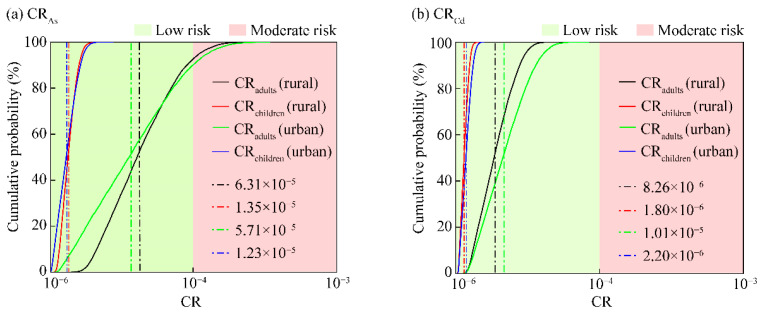
Probability distribution of the CR of HMs (the black, red, green, and blue vertical dashed lines represented the average CR values for adult (rural), children (rural), adult (urban), and children (rural), respectively).

As shown in the probability distribution of CR (Figure 4), the calculated average CR values of As in the urban and rural small wetlands were 6.31 × 10^−5^ and 5.71 × 10^−5^, respectively, for adults. The corresponding values for children were 1.35 × 10^−5^ and 1.23 × 10^−5^, respectively. All these values for As fall within the range of 1 × 10^−6^ to 1 × 10^−4^, indicating a low carcinogenic risk according to the classification standard. Meanwhile, the calculated average CR values of Cd in the urban and rural small wetlands were 1.01 × 10^−6^ and 8.26 × 10^−6^, respectively, for adults. The corresponding values for children were 2.20 × 10^−6^ and 1.80 × 10^−6^, respectively. All these values for Cd were less than 1 × 10^−6^, indicating a very low carcinogenic risk. Notably, at cumulative frequencies of 92.6% and 90.07%, the carcinogenic risks for adults from HMs in both the urban and rural small wetlands surpassed 1 × 10^−4^, at a moderate carcinogenic risk level.

**Figure 5:**
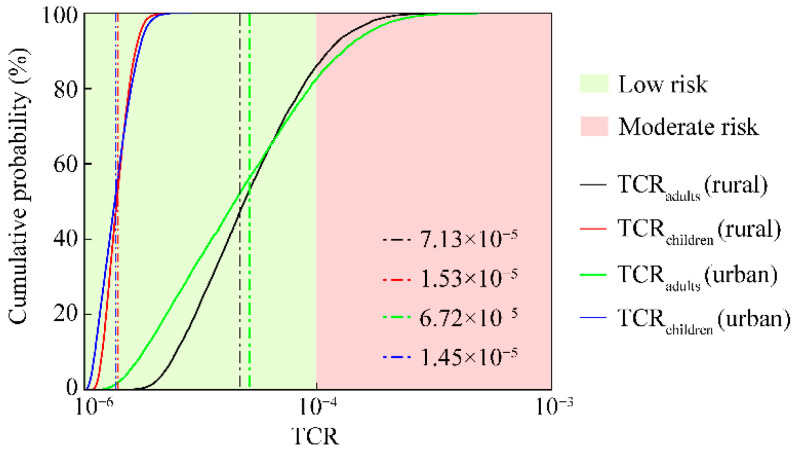
Probability distribution of the TCR of HMs (the black, red, green, and blue vertical dashed lines represented the average TCR values for adult (rural), children (rural), adult (urban), and children (rural), respectively).

The probability distribution of the TCR of HMs was illustrated in Figure 5. As shown in Figure 5, the probability distributions of TCR values, for adults and children, of HMs in the rural and urban small wetlands, namely, TCR_adults_ (rural), TCR_adults_ (urban), TCR_children_ (rural), and TCR_children_ (urban), were 7.13 × 10^−5^, 6.72 × 10^−5^, 1.53 × 10^−5^, and 1.45 × 10^−5^, respectively, at a low carcinogenic risk level. HMs in the rural small wetlands have relatively higher carcinogenic health risk than that of the urban small wetlands. Notably, at cumulative frequencies of 92.6% and 90.06%, the carcinogenic risks for adults in both the rural and urban wetlands exceeded 1 × 10^−4^, which indicated a moderate risk level. However, As was confirmed as the dominant carcinogenic risk factor in the small wetlands in the study area.

### 3.5. Sensitivity Analysis

**Figure 6:**
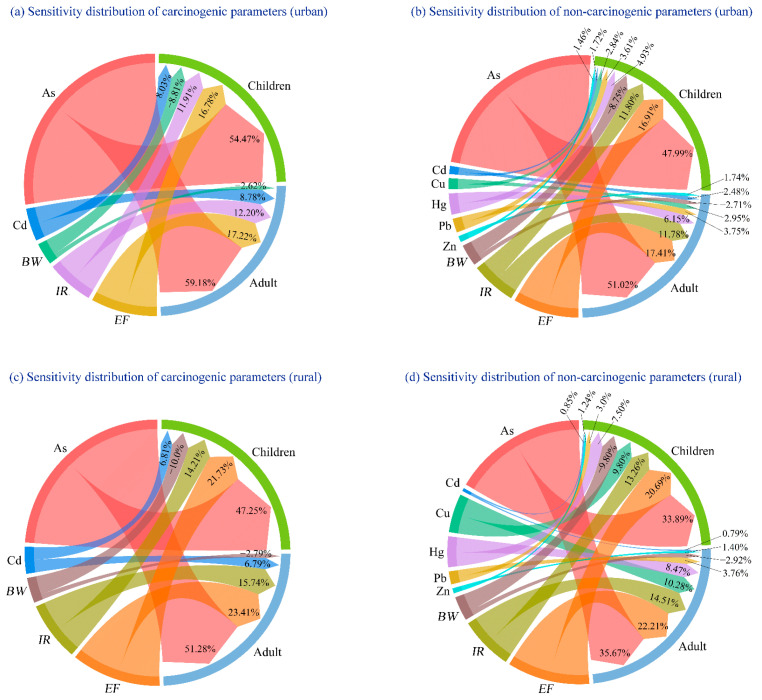
Sensitivity analysis of carcinogenic and non-carcinogenic health risks.

Sensitivity analysis was conducted to reveal the extent of influence of each parameter over the risk assessment results, with larger sensitivity values indicating a stronger impact. A positive sensitivity value denotes a positive correlation with the risk outcomes, while a negative value suggests the opposite (Figure 6).

As shown in Figure 6, based on the Monte Carlo simulations, all parameters, except for body weight (*BW*), showed a positive correlation with the risk assessment results. It is noteworthy that the impact of each parameter on the risk assessment results showed a similar trend in both the urban and rural wetlands. For non-carcinogenic risks of HMs in the small wetlands, the sensitivity values for adults can be ranked as follows: As (51.02%), *EF* (17.41%), *IR* (11.78%), Hg (6.15%), Pb (3.75%), Cu (2.95%), *BW* (−2.71%), Cd (2.48%), and Zn (1.74%). For children, the sensitivity values can be ranked as follows: As (47.99%), *EF* (16.91%), *IR* (11.80%), *BW* (−8.75%), Hg (4.93%), Pb (3.61%), Cu (2.84%), Cd (1.72%), and Zn (1.46%). Regarding the carcinogenic risks of HMs in the small wetlands, for adults, the sensitivity values can be ranked as follows: As (59.18%), *EF* (17.22%), *IR* (12.20%), Cd (8.78%), and *BW* (−2.62%). For children, the sensitivity values can be ranked as follows: As (54.47%), *EF* (16.78%), *IR* (11.91%), *BW* (−8.81%), and Cd (8.03%). These results indicate that As in the small wetlands was the primary factor influencing the results of health risk assessment, followed by the *EF* and *IR*, which significantly impact the results of health risk assessment. Notably, the health risk assessment results were more sensitive to BW for children, in comparison with adults.

## 4. Discussion

### 4.1. Concentration of HMs in the Overlying Water of Small Wetlands

The accurate estimation of contamination and potential risks of HMs in the small wetlands is prerequisites for wetland conservation and restoration. The investigation results of HMs in the small wetlands in the study area showed that the average concentration of Hg in the urban and rural small wetlands exceeded the Class II thresholds of the GB 3838-2002 by factors of 3.2 and 5.0, respectively, with the highest enrichment of Hg, whereas the average concentrations of other five HMs were lower than the threshold values. Based on the above analysis, the concentration of Hg in the small wetlands in the study area was significantly higher than the Class II threshold value of the national standard of China, indicating that Hg is particularly abundant compared to other investigated HMs in the small wetlands of the Urumqi.

Previous research [39] reported that the average concentrations of Hg in urban and rural soils in the Urumqi exceeded the corresponding background values by factors of 6.58 and 6.05 times, respectively, with the highest enrichment being in the Hg element. In general, soluble HMs in the sediments have the potential to migrate into wetland water through dissolution [53]. Therefore, the higher enrichment of Hg in the small wetlands in the study area may be linked to the high Hg concentration of soil. Hg is a globally distributed hazardous metal and could pose a significant health hazards [54]. Hg and its compounds were included in the “List of toxic and harmful water pollutants” of China [55]. Therefore, it is necessary to control its production so as to eliminate any potential threats to human health.

### 4.2. Comparison of HMs in the Overlying Water of Urban and Rural Small Wetlands

To highlight the differences in the contamination risk of HMs in the urban and rural small wetlands, the contamination degree and potential health risk level of HMs in the urban and rural small wetlands were compared. The obtained results of the contamination and potential health risk assessment revealed that, contrary to our expectations, the contamination degree and potential health risk level of HMs in the rural small wetlands were higher than that of the urban small wetlands. Specifically, the concentrations of Hg, As, Cu, Zn, Pb, and Cd in the rural small wetlands were surpassed the urban small wetlands by factors of 1.55, 1.54, 1.68, 1.89, 1.81, and 1.61, respectively.

However, intensive agricultural activities in rural areas may significantly contribute to higher concentrations of HMs in the rural small wetlands. Intensive agricultural practices often rely on various pesticides for different purposes [56], which can enter surface water and groundwater systems through various pathways [57]. The transport pathways of pesticides, particularly to small water bodies, are diverse, including overlying runoff and tile drainage due to soil leaching [58,59]. Rural small wetlands, owing to their strategic location in the agricultural landscape and lower dilution potential, along with their smaller water volume or discharge, may exhibit contaminant concentrations much higher than larger water bodies [60].

Secondly, the hydrological and ecological conditions of small wetlands also affect the presence of HMs in water. Certain stagnant or densely vegetated small wetlands can impede water flow, capturing and accumulating sediment-related contaminants, such as HMs [61]. Additionally, plants play a crucial role in absorbing potential contaminants, particularly during the growing season [62]. Plant roots can uptake HMs from the wetland sediments and transport them into the plant body. When plants decompose, the HMs they contain may enter the wetland water [63]. The richness of vegetation in the rural small wetlands in the study area was relatively higher than that of the urban small wetlands. In rural areas, however, the rural small wetlands remain stagnant, with limited or no water flow. The urban wetland samples collected in this study were mainly landscape small wetlands. These landscape wetlands may experience more frequent water replenishment, leading to higher water flow rates and quicker water cycle. This difference in hydrological and ecological conditions of small wetlands resulted in variations in the contamination levels and potential health risks of HMs in the urban and rural small wetlands. Consequently, contamination risk of HMs in the rural small wetlands in the study area were higher than that of the urban small wetlands.

### 4.3. Identification of the Main Risk Factor in the Overlying Water of Small Wetlands

**Figure 7:**
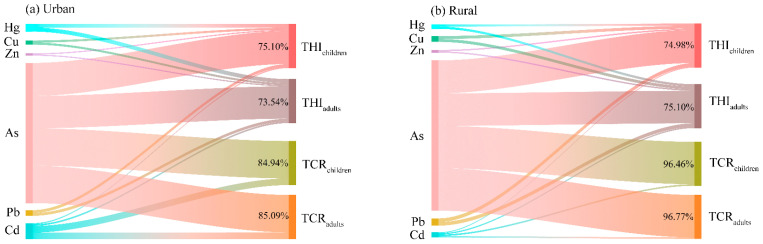
The relations between the health risks and HMs.

Identifying the relationship between health risks and HMs is pivotal for the prevention and control of HMs contamination of the small wetlands [64]. Quantifying the relationship between health risks and HMs can help establish risk mitigation strategies, which could best minimize the potential risks of HMs for human health [31]. As shown in Figure 7, the contribution rates of the investigated HMs to the potential health risks for the urban and rural small wetlands in the study area were similar. As was the dominant contributor for both the carcinogenic and non-carcinogenic health risk of the urban and rural small wetlands. Specifically, the contribution rates of As in the urban small wetlands to the THI were 75.10% and 73.54% for children and adults, respectively. Meanwhile, the contribution rates of As in the rural small wetlands to the THI were 74.98% and 75.10% for children and adults, respectively. Moreover, the contribution rates of As in the urban small wetlands to the TCR value were 84.94% and 85.09% for children and adults, respectively. Meanwhile, the contribution rates of As in the rural small wetlands to the TCR were 96.46% and 96.77% for children and adults, respectively. It further proves that concentration of As in wetland water has a significant impact on the results of the potential health risk assessment.

Arsenic is a hazardous metalloid that can accumulate in various water bodies and cause adverse human health risk [65]. As has the ability to move and transform in different ecosystems, and can threaten multiple organs through various routes, influenced by environmental bioprocesses [64]. Therefore, As contamination of water bodies has become a serious issue due to its high toxicity and bioaccumulation [66]. Results of this study emphasized that the potential human health risks caused by exposure to As in the small wetlands in the study area cannot be ignored.

## 5. Conclusions

In conclusion, this study analyzed the contamination risks of six HMs in the overlying water of urban and rural small wetlands, for the first time in the Urumqi of China. The Nemerow pollution index (NPI) and the US EPA health risk assessment model based on the Monte Carlo simulation were used for contamination and health risk assessment. Results revealed that the average concentrations of Hg in the overlying water of small wetlands surpassed the Class II thresholds of the Environmental Quality Standards for Surface Water (GB 3838-2002) by factors of 3.2 and 5.0, respectively, with high enrichment. The average concentrations of other five HMs were less than the corresponding threshold values. The individual contamination degree (*P_i_*) of HMs in the urban and rural small wetlands decreased in the order of *P*_Hg_ > *P*_Pb_ > *P*_Zn_ > *P*_As_ > *P*_Cu_ > *P*_Cd_, with high contamination of Hg. The NPI of HMs in the urban small wetlands fall into a moderate contamination level, whereas the NPI value of HMs in the rural wetlands belonged to a high contamination level. Results of the health risk assessment showed that the investigated HMs in the small wetlands cannot pose a non-carcinogenic risk for adults, but As may pose a non-carcinogenic health risk to children because the maximum HQ of As reached 2.38. As showed a low carcinogenic risk with CR values within the range of 1 × 10^−6^ to 1 × 10^−4^, and Cd showed the very low carcinogenic risk level with CR values lower than 1 × 10^−6^. Moreover, HMs in the rural small wetlands showed relatively higher contamination level and carcinogenic and non-carcinogenic health risk than that of the urban small wetlands. Finally, based on calculated health risks and sensitivity analysis, As was identified as the main health risk factor in the small wetlands in the study area.

The present study comparatively analyzed the contamination and potential health risks of HMs in the urban and rural small wetlands in arid zones. In the present study, water samples were collected only from small lakes and ponds because of the scarcity of other types of small wetlands (such as seasonal ponds, wet depressions, streams, springs, small rivers, and channels) under the extremely arid climate of this study area. However, all categories of natural and constructed small wetlands should be considered in future studies for obtaining a more comprehensive understanding of contamination risks HMs in small wetlands. Despite these limitations, the present study can clarify our understanding of the contamination risks of HMs in small wetlands in arid zones. The obtained results of this study could serve as valuable tools to identify the contamination levels and potential health risks, and the dominant risk factor in arid zone wetland ecosystems.

## Figures and Tables

**Table 1:** Classification of contamination degree of *P_i_* and NPI.

Class	Contamination Degree	*P_i_*	NPI
I	No contamination	*P_i_* < 0.7	NPI < 0.7
II	Slightly contamination	0.7 < *P_i_* ≤ 1	0.7 < NPI ≤ 1
III	Light contamination	1 < *P_i_* ≤ 2	1 < NPI ≤ 2
IV	Moderate contamination	2 < *P_i_* ≤ 3	2 < NPI ≤ 3
V	High contamination	*P_i_* > 3	NPI > 3

**Table 2:** The calculation parameters for CDI estimation.

Parameters	Meaning	Units	Children	Adults	References
*IR*	Ingestion rate	L/day	Mean = 1.25 SD = 0.3	Mean = 1.95 SD = 0.3	[33]
*EF*	Exposure frequency	days/year	Minimum = 180 Most probable = 350 Maximum = 360	Minimum = 180 Most probable = 350 Maximum = 360	[43]
*ED*	Exposure duration	year	2.5	26	[44]
*BW*	Body weight	kg	Mean = 20 SD = 0.15	Mean = 70 SD = 0.15	[45]
*AT_non-cancer_*	Averaging time of non-cancer risk	days	912.5	9490	[44]
*AT_cancer_*	Averaging time of cancer risk	days	25,550	25,550	[44]
*SA*	Skin surface area	cm^2^	7422	18,182	[33]
*KP*	Absorption coefficient	cm/h	10^−3^ for Hg, Cu, As, and Cd, 6 × 10^−4^ for Zn, and 10^−4^ for Pb		[46]
*T*_event_	Absorption coefficient	h/event	0.58	1	[46]
*EV*	Event frequency	event/day	1	1	[43]

Note: SD refers the standard deviation.

**Table 3:** The *RfD* and *SF* values of heavy metals.

HMs	*RfD_ing_* (µg/kg/d)	*RfD_derm_* (µg/kg/d)	*SF_ing_* (µg/kg/d)^−1^	*SF_derm_* (µg/kg/d)^−1^
Hg	0.3	0.024	/	/
As (for non-cancer)	0.3	0.123	/	/
As (for cancer)	/	/	1.5 × 10^−3^	3.66 × 10^−3^
Cu	40	12	/	/
Zn	300	60	/	/
Pb	1.4	0.42	/	/
Cd (for non-cancer)	0.5	0.005	/	/
Cd (for cancer)	/	/	6.1 × 10^−3^	3.8 × 10^−4^

**Table 4:** The Statistical summary of HMs in the overlying water of small wetlands.

Gradients	Statistics	Hg	As	Cu	Zn	Pb	Cd
Urban wetlands (*n* = 20)	Max (μg/L)	0.88	8.30	73.0	298.0	3.53	0.34
	Min (μg/L)	0.04	0.50	1.48	7.44	0.10	0.05
	Average (μg/L)	0.16	2.70	37.76	93.18	1.20	0.09
	SD (μg/L)	0.21	1.80	20.46	54.33	0.86	0.06
	CV *	1.29	0.67	0.54	0.58	0.72	0.73
	Standard-exceeding ratio (%)	80.0	0	0	0	0	0
Rural wetlands (*n* = 16)	Max (μg/L)	1.13	7.60	232.0	344.0	3.70	0.27
	Min (μg/L)	0.04	2.10	1.22	78.60	0.97	0.05
	Average (μg/L)	0.25	4.15	63.29	175.65	2.18	0.14
	SD (μg/L)	0.31	1.79	49.42	71.00	0.89	0.06
	CV	1.25	0.43	0.78	0.40	0.41	0.43
	Standard-exceeding ratio (%)	81.25	0	0	0	0	0
National standard (μg/L) **		0.05	50	1000	1000	10	5

Note: * CV = (SD of *x*)/(average value of *x*); ** Class II thresholds of the Environmental Quality Standards for Surface Water.

**Table 5:** The contamination level of HMs in the overlying water of small wetlands.

Gradients	Statistics	*P_Hg_*	*P_As_*	*P_Cu_*	*P_Zn_*	*P_Pb_*	*P_Cd_*	NPI
Urban wetlands (*n* = 20)	Max	17.60	0.17	0.07	0.30	0.35	0.07	12.62
	Min	0.80	0.01	0.00	0.01	0.01	0.01	0.58
	Average	3.21	0.05	0.04	0.09	0.12	0.02	2.31
Rural wetlands (*n* = 16)	Max	22.60	0.15	0.23	0.34	0.37	0.05	16.22
	Min	0.80	0.04	0.00	0.08	0.10	0.01	0.58
	Average	4.98	0.08	0.06	0.18	0.22	0.03	3.58

## Data Availability

Data will be available upon request to the corresponding author.
